# Cognitive Abilities, Monitoring Confidence, and Control Thresholds Explain Individual Differences in Heuristics and Biases

**DOI:** 10.3389/fpsyg.2016.01559

**Published:** 2016-10-13

**Authors:** Simon A. Jackson, Sabina Kleitman, Pauline Howie, Lazar Stankov

**Affiliations:** School of Psychology, The University of SydneySydney, NSW, Australia

**Keywords:** decision-making, cognitive abilities, confidence, control, heuristics, biases, metacognition

## Abstract

In this paper, we investigate whether individual differences in performance on heuristic and biases tasks can be explained by cognitive abilities, monitoring confidence, and control thresholds. Current theories explain individual differences in these tasks by the ability to detect errors and override automatic but biased judgments, and deliberative cognitive abilities that help to construct the correct response. Here we retain cognitive abilities but disentangle error detection, proposing that lower monitoring confidence and higher control thresholds promote error checking. Participants (*N* = 250) completed tasks assessing their fluid reasoning abilities, stable monitoring confidence levels, and the control threshold they impose on their decisions. They also completed seven typical heuristic and biases tasks such as the cognitive reflection test and Resistance to Framing. Using structural equation modeling, we found that individuals with higher reasoning abilities, lower monitoring confidence, and higher control threshold performed significantly and, at times, substantially better on the heuristic and biases tasks. Individuals with higher control thresholds also showed lower preferences for risky alternatives in a gambling task. Furthermore, residual correlations among the heuristic and biases tasks were reduced to null, indicating that cognitive abilities, monitoring confidence, and control thresholds accounted for their shared variance. Implications include the proposal that the capacity to detect errors does not differ between individuals. Rather, individuals might adopt varied strategies that promote error checking to different degrees, regardless of whether they have made a mistake or not. The results support growing evidence that decision-making involves cognitive abilities that construct actions and monitoring and control processes that manage their initiation.

## Introduction

Decision-making often depends on the use of mental shortcuts (heuristics), which avoid the need for overwhelming mental computation but can also bias our judgments under certain conditions (Tversky and Kahneman, 1974; Gilovich et al., 2002; Kahneman and Klein, 2009). Yet there are pervasive individual differences in the degree to which people exhibit these sorts of biases (Appelt et al., 2011). This presents an opportunity in areas such as the assessment of decision-making, which relies on the presence of reliable individual differences. However, it also presents complexities. For example, this means that a one-size-fits-all approach will fail when it comes to predicting conditions that elicit heuristics, designing effective decision aids, and so on. Therefore, to support solutions for these opportunities and issues, this paper will aim to investigate whether individual differences in heuristics and biases (H&B) can be explained by three decision-relevant constructs: cognitive abilities, monitoring confidence, and control thresholds.

### Heuristics and Biases

To provide some context, we will first examine a well-known task designed to elicit the use of a heuristic that leads to a biased judgment: the bat and ball problem from Frederick’s (2005) Cognitive Reflection Test:

Together a bat and a ball cost $1.10.

The bat costs $1 more than the ball.

How much does the ball cost?

Most people respond that the answer is 10 cents (Frederick, 2005). Some mental shortcut (heuristic) is thought to construct this response quickly and seemingly effortlessly, without the need for mental calculations. A simple verification will show that the answer is five cents, however. Thus, items in the Cognitive Reflection Test elicit heuristically constructed but incorrect responses that must be checked and corrected. Indeed, many of the tasks used to establish that, and further investigate how we use heuristics when making decisions are similar to this. That is, most H&B tasks present a question that allows for a normatively correct response, but which people often avoid.

### Individual Differences

Not long after their emergence, it became apparent that individuals varied in their susceptibility to committing errors and biases elicited by H&B tasks (e.g., Stanovich and West, 1998a,b,c). For example, while many people respond “10 cents” to the problem above, some respond correctly. Individuals’ performance on a range of H&B tasks has now been found to correlate in a weak to moderate fashion (e.g., Stanovich and West, 2000; Parker and Fischhoff, 2005; Bruine de Bruin et al., 2007; West et al., 2008; Stanovich et al., 2012; Teovanović et al., 2015). An ongoing challenge being addressed by this literature has been to explain the nature and source of this covariance.

There have been two prominent approaches for explaining these individual differences in H&B tasks. Researchers originally hypothesized that a single rationality-like construct might be the source. The first approach was to therefore model covariance among H&B tasks via factor analysis to extract common sources underlying performance. Factor analyses of H&B tasks tend to yield solutions with two or more factors that are difficult to interpret within and between studies, however (Parker and Fischhoff, 2005; Bruine de Bruin et al., 2007; Weaver and Stewart, 2012; Teovanović et al., 2015). Furthermore, these sorts of psychometric studies demonstrate that H&B tasks tend to have relatively low reliability estimates. It is clear from this work that H&B tasks are not collectively and consistently tapping a single, general, and desirable construct.

The second approach has involved seeking correlates/predictors of individual’s H&B task performance on the basis of dual-process theories. According to these theories, two broad categories of cognitive processes construct judgments and actions (Evans and Stanovich, 2013 for a review). Type 1 processes are automatic and tend to rely on knowledge structures acquired via learning. They include processes like associative and constructive intuition (Glöckner and Witteman, 2010; Evans and Stanovich, 2013). Type 1 processes are therefore the typical source of heuristic responses that lead to errors on H&B tasks. Type 2 processes are deliberative and effortful mental operations. A classic example is fluid reasoning ability (Gf; Carroll, 1993; Stankov, 2000; McGrew, 2005, 2009). Gf is defined as “deliberate and controlled mental operations to solve novel problems that cannot be performed automatically” (McGrew, 2009, p. 5). Their reliance on working memory and controlled attention impose limits of their processing capacity (Evans and Stanovich, 2013; Shipstead et al., 2014). Such Type 2 abilities are the source of accurate responses on many H&B tasks. Investigated predictors of H&B tasks, therefore, tend to relate to Type 2 abilities or constructs that help shift decision makers from erroneous Type 1 to more accurate Type 2 thinking.

**FIGURE 1:**
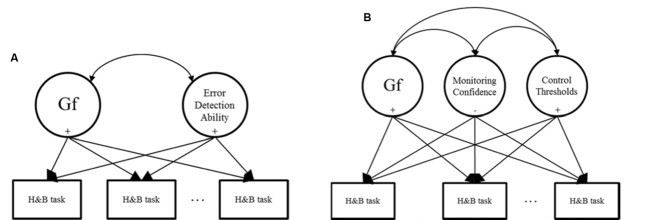
**Models used for predicting individual differences in heuristic and biases (H&B) tasks. (A)** Represents existing approaches; **(B)** represents the approach adopted in this research.

Stanovich and West (2008) proposed that Type 1 heuristic errors on tasks like the Cognitive Reflection Test must be detected so that Type 2 processes like Gf can become engaged. By this account, individuals with stronger Type 2 cognitive abilities and who are better able to detect errors of judgment will perform better on H&B tasks. Researchers have therefore tested models such as that shown in **Figure 1A**.

Adopting this approach, researchers measure individuals’ performance on a H&B task and predict the scores with cognitive abilities (e.g., Gf) and variables related to error detection (e.g., West et al., 2008; Toplak et al., 2011; Del Missier et al., 2012; Thompson and Johnson, 2014). In these studies, individuals’ ability to detect errors tends to be measured via executive control tasks in which they must suppress proponent and inaccurate responses in response time tasks. Alternatively, individuals are asked to self-report their tendency to engage in or enjoy Type 2 cognitive processing via self-report measures. Such studies typically find that Gf and error detection-like constructs are significant positive predictors of performance on H&B tasks in the manner proposed above.

Given these findings, we will assess here two ways to obtain greater information about individual differences in H&B tasks like the Cognitive Reflection Test. The first will be to model changes in covariance among H&B tasks. Rather than factor analyzing all tasks together or regressing each task independently on predictors, we will adopt a new approach. Specifically, we will regress individuals’ H&B task scores on a set of predictors in a single Structural Equation Model, and allow their residuals to correlate freely. If the predictors are generally underlying performance on the H&B tasks, then we should observe more than just the significant regression coefficients that are found when each H&B task is used in separate regression models. More specifically, the residual covariance among the H&B tasks in a single SEM (i.e., the correlated error terms after being regressed on the predictors) should be considerably lower than their zero-order correlations, and possibly null. If the predictors are accounting for task-specific variance only, then we should observe significant regression coefficients, but little change from the zero-order to the residual correlations among the H&B tasks.

Our second addition will be to disentangle the notion of error detection into distinct aspects of monitoring and control. That is, model monitoring and control variables separately, and use these as predictors of H&B performance instead of a single error detection construct. Overall, our goal here is to build on previous work by extending theory and measurement surrounding error detection as a predictor of individuals’ H&B task performance. **Figure 1B** shows our proposed model. The following section will describe the monitoring and control aspects, and their inclusion as independent predictors of individuals’ H&B performance is a novel approach in the literature.

### Monitoring and Control

**FIGURE 2:**
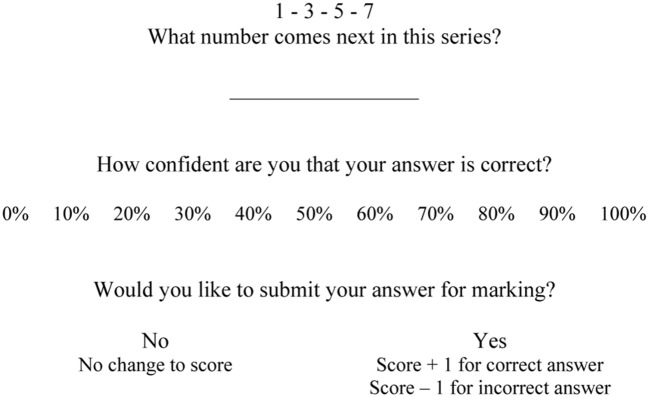
**Example item from the Number Series Test**.

Monitoring processes accumulate evidence in favor of judgments and actions during their construction (via Type 1 or Type 2 processes; Flavell, 1979; Koriat, 2012; Ratcliff and Starns, 2013). Confidence refers to this feeling or experience of evidence. For example, a clinician with excellent monitoring would be very confident for correct diagnoses but not at all confident for incorrect diagnoses. Confidence is known to be a stable individual differences trait and a predictor of action initiation. For example, Jackson et al. (2016a,b) had participants answer test items, indicate answer confidence, and decide whether to submit the answer for marking. **Figure 2** shows an example item.

Individuals’ trait Confidence is computed as the average of their confidence ratings for all test items. Jackson et al. (2016a,b) found that individuals with higher trait Confidence consistently submitted more answers for marking (correct and incorrect) than individuals with lower Confidence.

**FIGURE 3:**
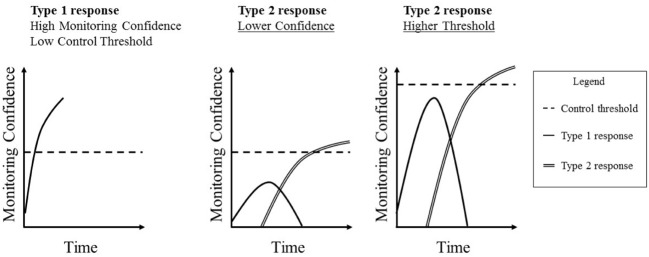
**Examples of how monitoring confidence accumulates toward a control threshold over time leading to a Type 1 or Type 2 response**.

Control processes determine the threshold point of confidence at which an action that is being considered will be initiated (Koriat and Goldsmith, 1996; Ratcliff and McKoon, 2008; Ackerman, 2014; White et al., 2015)^1^. For example, a clinician might treat a patient only once s/he is more than 90% confident in the diagnosis (e.g., Pauker and Kassirer, 1980; Djulbegovic et al., 2014). Jackson et al. (2016a,b) also assessed this by computing the threshold of confidence at which individuals would submit their answers (referred to in their work as the Point Of Sufficient cerTainty, or POST). Controlling for their confidence, individuals with a higher control threshold submitted fewer answers (correct and incorrect) for marking. Setting a high threshold, therefore, represents a risk-averse strategy, as the decision maker has more time to process potential actions while confidence is being accumulated. A higher threshold increases the likelihood of detecting errors, but the cost is that it may also increase the number of missed opportunities to submit correct answers, though it can never decrease that number (e.g., Koriat and Goldsmith, 1996). Setting a lower threshold will decrease (or maintain) the proportion of missed opportunities, but at the cost of also decreasing the likelihood of detecting errors. Including monitoring confidence and control thresholds in the model provides a more detailed account of how we manage the initiation of actions than the error detection theory proposed earlier. Shown in **Figure 3**, we can conceive the process of answering an H&B question as Type 1 and Type 2 processes occurring over time. Type 1 heuristics quickly generate an incorrect response, and, given the fluency with which this answer is generated, monitoring confidence will quickly rise in support of it. If this level of confidence reaches the control threshold, this initial response will be given. If, however, this does not happen, Type 2 processes will start to check the original response and, possibly, generate the accurate response. This will likely cause confidence in the initial response to decline, and confidence in the Type 2 response to increase until it reaches the control threshold. Note that this does not describe the general state of affairs. Type 1 responses are not always incorrect or faster than Type 2 responses. This just tends to apply for typical H&B tasks. What should generally apply, however, is that a response is chosen when confidence reaches a threshold.

From this perspective, it is likely that an individual who tends to be confident will often be confident enough in their initial and incorrect response to give it without further consideration. The same logic applies to an individual who tends to set a low control threshold. However, an individual who tends to be less confident, and/or hold a higher control criterion, will be more likely to engage in Type 2 processing. Provided that they also possess and utilize the necessary Type 2 cognitive abilities, individuals who tend to experience low monitoring confidence and/or hold high control thresholds will more probably reach the correct answer. Thus, from an individual differences perspective, individuals with relatively low monitoring confidence, a high control threshold, and strong cognitive abilities (like Gf) should do best on H&B tasks.

Although similar, the monitoring and control view differs from the error detection perspective in an important way. By the latter, the ability to detect errors should differ between individuals. Such a skill should be evidenced by monitoring confidence discriminating between good and bad judgments/actions. However, the literature on these sorts of monitoring skills has revealed that they tend to be unreliable as individual differences variables (Stankov and Crawford, 1996; Jackson and Kleitman, 2014; Stankov et al., 2014). That is while people tend to discriminate between accurate or inaccurate judgments, in general, individuals tend not to be better or worse than each other at doing this. Such results speak against the theory that individuals are better or worse than each other at “detecting” errors, independent of intelligence, skills, etc.

Individuals do differ consistently in their overall level of monitoring confidence and control threshold, however. Individuals vary in their overall average level of confidence (Pallier et al., 2002; Kleitman and Stankov, 2007; Stankov et al., 2014). Similarly, individuals tend to vary in the control threshold they set within a given context (Jackson et al., 2016a,b). These results suggest that people adopt different strategies for checking errors of judgments. As described above, low-confidence-high-threshold individuals would be more likely to check for errors than high-confidence-low-threshold individuals, regardless of errors being made or not. The low-confidence-high-threshold combination should, therefore, lead to the highest performance level on H&B tasks, which do elicit automatic errors.

We will examine one implication of this theoretical difference: that monitoring confidence and, in particular, control thresholds link a broader range of decision constructs than the ability to detect error. For example, a wide class of Bias tasks are gambling measures that assess risk and ambiguity preference. These differ from tasks like the Cognitive Reflection Test as the final score indicates a difference in preference rather than performance. The notion of error detection cannot account for how or why these sorts of tasks should relate to the broader range of H&B tasks. Such gambling biases should relate, however, to control thresholds. As mentioned above, individuals who set a higher threshold are adopting a more risk-averse strategy than individuals setting lower thresholds. This is because adopting a high control threshold means that you will not make a decision until you are sufficiently certain that the decision is a good one. For example, holding a threshold of say 90–100% means that you must be very certain of a decision before initiating it. Therefore, adopting a high control threshold is conceptually similar to having a low preference for risky, uncertain, or ambiguous outcomes. Adopting a low threshold such as 30–40%, however, means that you are willing to make a decision despite great uncertainty. This is akin to having a high preference for risk, uncertainty, or ambiguity. As such, control thresholds provide a theoretical basis for expecting a negative correlation between individuals’ preference for risk on gambling tasks and performance on H&B tasks. Thus, in addition to employing typical H&B tasks, we will expand our dependent variables to include measures assessing biases for uncertain and ambiguous outcomes (Risky Gambles and Ellsberg Paradox). Individuals holding higher control thresholds are expected to select more certain options (meaning fewer uncertain and ambiguous options) on the gambling tasks than individuals with lower thresholds.

### Aims and Hypotheses

To summarize, our goal is to investigate the combined predictive relations of individuals’ fluid reasoning abilities (Gf), monitoring confidence, and control thresholds with a range of H&B tasks. We will be extending previous work in two ways. First, by modeling the residual covariance among the H&B tasks to determine the extent to which cognitive abilities, monitoring confidence, and control thresholds account for common variance. The degree to which the zero-order correlations among the H&B tasks are reduced will indicate the degree to which their covariance can be explained by cognitive abilities, monitoring confidence, and control thresholds. Second, by independently accounting for monitoring and control variables, as an extension of previous theories proposing that the ability to detect errors differs between individuals. We hypothesize that:

H1. (a) Individuals with higher Gf will perform better on H&B tasks than those with lower Gf.
(b) Individuals with lower monitoring confidence will perform better on H&B tasks than those with higher monitoring confidence.
(c) Individuals with a higher control threshold will do better on H&B tasks than individuals with a lower threshold.
H2. Individuals with a higher threshold will select more certain options in the gambling tasks than individuals with a lower control threshold.
H3. After accounting for variance predicted by Gf, monitoring confidence, and control thresholds, the residual correlations among the H&B tasks will be weaker than their zero-order correlations, and possibly null.

These hypotheses will be tested via structural equation modeling to account for shared covariance among the variables. Before these final tests, however, inference will be made, where possible, on the basis of the zero-order correlation matrix.

## Materials and Methods

### Participants

In return for partial course credit, 250 first year psychology students at the University of Sydney participated in the study (*N* = 250, 158 female, 92 male, *M*_age_ = 20.46 years, age range: 17.41–60.65 years). Participants completed all tests below within a larger battery, which included measures of executive function^2^.

### Measures

#### Fluid Cognitive Abilities (Gf) and Monitoring Confidence

##### Raven’s Advanced Progressive Matrices (short version)

For each item, participants see a 3 × 3 matrix of abstract figures following a pattern (Raven, 1938–1965). The bottom right figure is blank, and participants chose which of eight alternatives completes the pattern. Accuracy is a gold standard measure of Gf. As shown in **Figure 2**, each item was accompanied by a confidence rating and decision to submit the answer for marking or not. Participants were instructed to try and maximize their overall test score. To reduce the duration of the study and fatigue, a subset of 12 of the standard 36 items were selected *a priori* for administration in this study, on the basis of previous research using Australian undergraduates (Jackson and Kleitman, 2014; Jackson et al., 2016a,b). This selection is consistent with our own and others psychometric analyses of previous data using this cohort as these items have been found to be suitable for assessing a unidimensional reasoning construct (Waschl et al., 2016). For each participant, mean Accuracy and Confidence scores are computed across all items.

##### Esoteric Analogies Test

For each item, participants must complete an analogy such as “CONSTELLATION is to STAR as ARCHIPELAGO is to: ISLAND (correct answer), PENINSULA, CONTINENT, COUNTRY?” (Stankov, 1997). Accuracy depends on both knowledge and Gf, as the participant needs to comprehend the words and then apply the relationships. Confidence ratings and the decision to submit answers for marking follow each item. On the basis of previous research (Jackson and Kleitman, 2014; Jackson et al., 2016a,b), 18 items were selected from the total 24. For each participant, mean Accuracy and Confidence scores are computed accross all items.

##### Number Series Test

For each item, participants type the number they think completes a series such as 2 – 4 – 6 – 8 – ? (correct answer: 10). Participants are permitted to use a calculator to remove the influence of numerical ability. Accuracy, therefore, depends on Gf, as the participant needs to determine the rules underlying the series. Twelve items are automatically generated as recommended by Arendasy and Sommer (2012). All participants received items of equivalent numbers of rules, numbers of operations, periodicity, and complexity to each other. Confidence ratings and the decision whether to submit answers for marking accompany each item. For each participant, mean Accuracy and Confidence scores are computed accross all items.

#### Control Thresholds

##### Medical Decision-Making Test

In this test, participants adopt the role of a specialist in the Alpha virus (Jackson and Kleitman, 2014; Jackson et al., 2016a,b). Participants are instructed that the Alpha virus can occur in a regular form or one of three mutations (A1, A2, and A3). After having 3 min to memorize how nine symptoms are associated with each form, participants diagnose patients presenting with two symptoms each. For time reasons, participants completed a half-form version of this test with 16 rather than 33 patients. A confidence rating and a decision to treat the patient or request a blood test accompanies each diagnosis. Participants are instructed to save as many lives as possible by treating correctly diagnosed patients or blood test misdiagnosed patients. In this research, this test was used to obtain a measure of individuals’ control threshold: the point of confidence at which individuals were sufficiently certain in their diagnosis to treat a patient. The threshold was calculated by regressing the decision (treat or blood test) on paired confidence ratings in a logistic regression for each participant (Jackson et al., 2016a for details).

##### Financial Decision-Making Test

This test followed a similar format to the Medical Decision-Making Test, but in a financial setting, and was developed for this experiment. In each item, participants choose a product in which to invest. The goal is to select the product likely to yield the greatest return. Each item presents a table of six products and five features. Each feature carries an expected return. For example, feature *A* might have a 30% chance of returning $2000 and a 70% chance of returning nothing. Each product has a different feature combination. Two of the six products yield the greatest expected return, with one being based on more risky features than the other. A confidence rating and a decision to invest in the chosen product or spend money on a research team to help find the best product follow each product choice. In this research, this test was used to obtain a second measure of individuals’ control thresholds: the point of confidence at which individuals were sufficiently certain in their product choice to invest.

#### Heuristics and Biases

##### Cognitive Reflection Test

This test comprises of numerical problems such as the bat and the ball problem described in the section “Introduction.” Here we used an expanded 7-item version of this test (Toplak et al., 2014) that includes Frederick’s (2005) three original items. The longer test version was used to ensure satisfactory psychometric properties (Toplak et al., 2014). Participants type their response for each item. The final score is computed as the number of questions out of seven answered correctly.

##### Applying Decision Rules

For each item in this test, participants must determine which DVD player(s) meet the rules laid out by a potential consumer (Bruine de Bruin et al., 2007). Each item includes five DVD players that are rated from very low (1) to very high (5) on four attributes (e.g., picture quality). The rules are selected to elicit heuristics related to elimination by aspects, satisficing, lexicographic, and equal weights rules (Payne et al., 1993). These must be overcome to determine the correct answer deliberatively. For example, “Lisa wants the DVD player with the highest average rating across features,” elicits the equal weights rule. The test includes 10 items of this type, and the final score is computed as the number answered correctly.

##### Consistency in Risk Perception

This task assesses the ability to follow probability rules. Participants rate the probability from 0% (no chance) to 100% (certainty) that they will experience various events (Bruine de Bruin et al., 2007). For example, “What is the probability that you will die in a terrorist attack in the next year”? Participants rate 10 events in two time frames (20 ratings all up): that the events will occur in the next year and the next 5 years. Each event pair is scored as correct if the probability assigned to the next year version is no greater than the probability assigned to the 5-year version. In each time frame, three items are nested subsets of others. For example, dying in a terrorist attack is nested within dying as a result of any cause. Each nested item receives an additional point if it is assigned a probability no greater than its relevant superset. Further, in each time frame, two items are complementary to others. For example, getting in a car accident is complementary to being accident free. Each of these items receives an additional point if its assigned probability is equal to 100 minus probability assigned to the complementary. The final score is a mark out of 20 possible points: 10 time-frame pairs, six nested pairs, and four complementary pairs.

##### Resistance to Framing

This task assesses whether irrelevant features of the problem description influence an individual’s value assessment. Participants rate their preference for (1) definitely option A to (6) definitely option B, in seven scenarios. Each scenario presents a risky option and a sure-thing option (Bruine de Bruin et al., 2007). For example, one item states that the U.S. is preparing for the outbreak of an unusual disease, which is expected to kill 600 people. Participants must indicate their preference for adopting program A, which will save 200 people for sure, or program B, which has a 1/3 chance of saving all 600 people, and a 2/3 chance of saving none. Participants complete all seven scenarios twice. At the beginning of the test battery, scenarios are framed in terms of gains such as the example above which frames outcomes in terms of people saved. At the end of the test battery, the scenarios are presented with equivalent options but framed in terms of losses. For example, the options for the disease scenario above would be (A) 400 people die for sure, (b) 1/3 chance of 400 people dying, and 2/3 chance of 600 dying. The absolute difference between the sum of the ratings for the positive and sum of the ratings for negative versions evidences performance. For this variable, however, scores closer to zero indicate better performance. Here, this value is reverse scored by subtracting it from 42 (the max score if all ratings were 6) so that higher scores indicate better Resistance to Framing.

##### Resistance to Sunk Costs

This task assesses the ability to ignore prior, but now inconsequential costs when making a decision. Participants read 10 scenarios that present a prior cost followed by one option that ignores the cost (the normatively correct option) and one that does not (the sunk cost option). They then rate whether they would be more likely to continue with (1) the sunk cost option or (6) the normatively correct option. For example, one scenario states that you’ve just ordered a dessert with chocolate and ice cream after eating a large meal. You have a few bites and find that you are full and would rather not eat any more. The participant rates whether they would be more likely to eat more (sunk cost) or stop eating. The final score is the mean rating with the highest score of six representing the best Resistance to Sunk Costs.

##### Risky Gambles

This task assesses a preference for risky over certain alternatives (Lauriola et al., 2007). Participants are instructed to adopt the role of a manager deciding between buying and selling contracts. For each item, participants select one of two contracts. One contract offers a certain outcome such as a 100% chance of gaining $10,000. The other offers a risky but normatively equivalent outcome such as a 25% chance of gaining $40,000 and 75% chance of gaining nothing. Participants decide upon 20 contracts. These comprise of four expected outcomes of $1000 or $20,000 being lost or gained, combined with five risky equivalents matched to 2, 25, 50, 75, and 98% of the expected value. The final score is the number of risky alternatives out of 20 selected.

##### Ellsberg Paradox

This task assesses a preference for ambiguous (unknown probabilities) over a known risk (Ellsberg, 1961; Lauriola et al., 2007). Each item presents the participant with two urns each containing 100 black and white balls in total. The distribution of black and white balls is unknown in one urn (the ambiguous urn) and known in the other. The participant must choose the urn they believe would have the best chance of drawing a white ball on the first draw. There are nine items, with the number of white balls in the known risk urn incrementing from 10 to 90 out of 100. The final score is the number of ambiguous urns out of nine selected.

#### Personality

##### Mini IPIP

This is a 50-item self-report measure of the Big-5 Personality dimensions: Intellect, Conscientiousness, Extraversion, Agreeableness, Emotional Stability (Donnellan et al., 2006). Results for these personality dimensions are not reported here because, consistent with much of our own previous research (e.g., Kleitman, 2008; Jackson and Kleitman, 2014; Stankov et al., 2014; Jackson et al., 2016a,b), they did not show any meaningful association with the on-task decision-making and on-task metacognitive variables. Consistent with these results, Burns et al. (2016) demonstrated that, in contrast to self-report measures of confidence, on-task measures of confidence do not meaningfully relate with self-report personality measures. Thus, maintain our focus on the on-task measures, results for this Personality scale will not be reported.

### Procedure

Participants signed up to participate over the Internet and received a URL to access these tests (within a larger battery) and complete them online. A notification appeared before each test reminding participants to take a short break. However, they were required to progress through the entire protocol in a single session. That is, they could have a short break after completing each test, but not exit the Internet browser and return at a later time or different place. Most participants completed the entire battery within a single 2-h period. Participants received partial course credit upon completion of the protocol but were not otherwise incentivized. Although this is a potential concern, previous research in our lab has revealed negligible differences between online participation (as done here) and in-person lab participation (Jackson, 2016).

## Results

### Descriptive Statistics

**Table 1** shows the descriptive statistics for all variables. Internal consistency estimates were computed as Cronbach’s alpha (Cronbach, 1951) for all variables except the control threshold. For the control threshold, internal consistency was computed on the basis of an Odd/Even item split correlation corrected using the Spearman-Brown Formula (Stankov and Crawford, 1996; Jackson et al., 2016a,b).

**Table 1:** Descriptive statistics for all variables.

	Mean	*SD*	Min	Max	IC
**Cognitive Abilities (Gf)**				
Advanced Progressive Matrices Accuracy (%)	57.30	20.75	8.33	100.00	0.68
Esoteric Analogies Test Accuracy (%)	62.20	18.71	16.67	100.00	0.74
Number Series Test Accuracy (%)	76.66	16.73	33.33	100.00	0.58
**Monitoring Confidence**				
Advanced Progressive Matrices Confidence (%)	65.84	15.52	30.83	100.00	0.84
Esoteric Analogies Test Confidence (%)	67.04	18.98	0.00	100.00	0.94
Number Series Test Confidence (%)	81.14	14.53	33.33	100.00	0.84
**Control Thresholds**				
Medical Decision-Making Test Threshold (%)	52.85	16.01	10.15	96.67	0.80
Financial Decision-Making Test Threshold (%)	44.20	15.79	0.31	92.64	0.56
**Heuristics/biases**				
Cognitive Reflection Test (/7)	2.07	1.85	0.00	7.00	0.70
Applying Decision Rules (/10)	5.69	2.12	1.00	9.00	0.60
Consistency in Risk Perception (/20)	15.32	2.05	8.00	20.00	0.50
Resistance To Framing (/42)	37.64	4.14	19.00	42.00	0.61
Resistance to Sunk Costs (/6)	4.12	.66	2.10	6.00	0.39
Risky Gambles (/20)	8.95	3.22	0.00	17.00	0.63
Ellsberg Paradox (/9)	3.03	1.25	0.00	5.00	0.43

*IC, internal consistency estimate*.

Descriptive statistics for all measures were typical for an Australian undergraduate sample. Mean Accuracy in the Gf tasks indicated a suitable level of difficulty (range = 57.30–76.66%). Confidence levels for the Gf tasks (range = 65.84–81.14%) and control thresholds from the other decision tasks (52.85 and 44.20%) were also within a typical range comparing to previous work on similar cohorts (Jackson et al., 2016a,b). For these variables, internal consistency estimates were poor for two variables: Accuracy in the Number Series Test (0.58), and the control thresholds for the novel Financial Decision-Making Test (0.56). The likely reason for this is that both of these tests were relatively short and newly developed. However, internal consistency estimates for the remaining variables above were good (range = 0.68–0.94).

Mean scores for the performance-based H&B tasks were in the typical range (e.g., Bruine de Bruin et al., 2007). However, internal consistency estimates for two of the H&B tasks were unacceptably low: Resistance to Sunk Costs task (0.39) and the Ellsberg Paradox (0.43). These low estimates indicated that we could not interpret the scores for these tasks in a meaningful fashion (Cronbach, 1951). We, therefore, removed these tasks from further analyses. In line with previous research (Bruine de Bruin et al., 2007; Del Missier et al., 2012; Weller et al., 2012), internal consistency estimates for the remaining H&B tasks were low but acceptable for research purposes (range = 0.50–0.70).

**Table 2** shows the correlations between all retained variables. The Gf Accuracy variables all correlated in a moderate fashion with each other. Confidence scores from the cognitive ability tests also correlated with each other and Accuracy scores. In the line with recent work using the same tests (Jackson et al., 2016a,b), Confidence and Accuracy scores from the same test had somewhat higher correlations than those observed with achievement tests in the past (Stankov et al., 2012). Following this recent work, test-specific correlations were managed in the forthcoming structural equation modeling by allowing residuals of the Accuracy and Confidence variables from the same test to correlate. The two control threshold scores correlated positively with each other and weakly with the Accuracy and Confidence variables.

**Table 2:** Correlations between retained variables.

	1	2	3	4	5	6	7	8	9	10	11	12
1. APM Accuracy												
2. EAT Accuracy	0.35^∗^											
3. NST Accuracy	0.41^∗^	0.35^∗^										
4. APM Confidence	0.61^∗^	0.21^∗^	0.31^∗^									
5. EAT Confidence	0.16^∗^	0.56^∗^	0.12	0.34^∗^								
6. NST Confidence	0.30^∗^	0.24^∗^	0.72^∗^	0.42^∗^	0.29^∗^							
7. MDMT Threshold	0.05	-0.13	-0.07	0.18^∗^	0.10	0.06						
8. FDMT Threshold	0.03	-0.21^∗^	-0.07	0.25^∗^	0.04	0.04	0.40^∗^					
9. Cognitive Reflection Test	0.49^∗^	0.38^∗^	0.43^∗^	0.29^∗^	0.23^∗^	0.30^∗^	0.01	0.15				
10. Applying Decision Rules	0.34^∗^	0.44^∗^	0.45^∗^	0.11	0.19^∗^	0.24^∗^	-0.09	-0.05	0.43^∗^			
11. Consistency in Risk Perception	0.16^∗^	0.11	0.11	0.02	0.01	0.05	-0.06	-0.08	0.14^∗^	0.22^∗^		
12. Resistance to Framing	0.06	0.13^∗^	-0.10	-0.09	0.06	-0.18^∗^	-0.09	-0.02	0.01	0.13^∗^	0.00	
13. Risky Gambles	-0.02	-0.04	-0.03	-0.09	-0.07	-0.03	-0.07	-0.18^∗^	-0.17^∗^	-0.04	0.05	0.06

*^∗^p < 0.05.*

*APM, Raven’s Advanced Progressive Matrices; EAT, Esoteric Analogists Test; NST, Number Series Test; MDMT, Medical Decision-Making Test; FDMT, Financial Decision-Making Test.*

In line with previous work (Parker and Fischhoff, 2005; Bruine de Bruin et al., 2007; Del Missier et al., 2012), the H&B tasks correlated with each other in a weak to moderate fashion. Applying Decision Rules and the Cognitive Reflection Test related most strongly to the other variables, with these tasks correlating significantly with almost all other tasks. Consistent with Hypothesis 1a, Gf Accuracy scores correlated, by-and-large, positively with the H&B tasks. Consistent with Hypothesis 1b, confidence scores also correlated positively and significantly with some H&B tasks. Though, these were not as strong as the correlations of the H&B tasks with the Accuracy scores, and some of these correlations were negative (but weak). Although we had not hypothesized these positive correlations, the strong positive correlations of Confidence with Accuracy scores was likely the reason for this. Hypothesis 1c, however, was not supported: The control threshold variables did not correlate significantly with the performance-based H&B tasks. It did, however, correlate significantly and negatively with the number of risky alternatives selected on the gambling task, consistent with Hypothesis 2. This matrix was submitted to structural equation modeling to more clearly investigate the hypotheses.

### Structural Equation Modeling

A structural equation model was constructed in two steps to investigate the hypotheses.

#### Step 1

**FIGURE 4:**
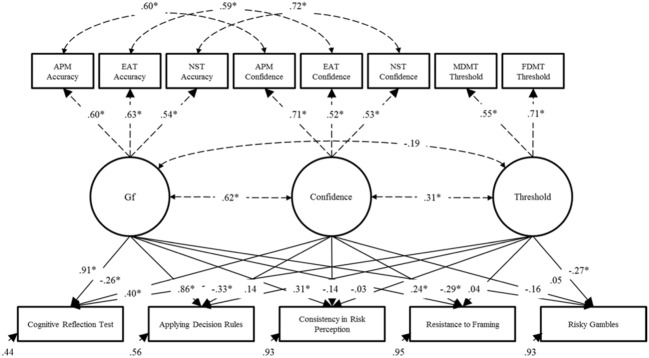
**Results of the final structural equation model described in Step 2**. Dashed lines represent fixed parameters whose values and significance levels were determined in Step 1 of the model. ^∗^*p* < 0.05. APM, Raven’s Advanced Progressive Matrices; EAT, Esoteric Analogists Test; NST, Number Series Test; MDMT, Medical Decision-Making Test; FDMT, Financial Decision-Making Test.

The model specified in Step 1 reflects consistent modeling decisions made in our previous work (Jackson et al., 2016b). Gf, monitoring confidence and control threshold factors were defined and allowed to covary freely. As intended, the Gf factor was defined by Accuracy scores from the three cognitive tests (APM, EAT, and NST). It was possible to compute monitoring confidence and control thresholds for these and the other tests (MDMT and FDMT). However, as statistically dependent variables cannot be included in the same SEM, the monitoring confidence factor was defined by confidence scores from the cognitive tests (APM, EAT and NST), and the control threshold factor on the basis of the MDMT and FDMT. Given the strong method correlations, residuals between Accuracy and Confidence variables from the same test were allowed to covary. Model fit was good: χ^2^_14_ = 28.87, *p* = 0.01, χ^2^/df = 2.06, CFI = 0.98, TLI = 0.95, RMSEA = 0.07 [0.03, 0.10], SRMR = 0.06. All factor loadings and residual correlations among the test-specific Accuracy and Confidence scores were strong and significant (*p* < 0.001). In line with previous research (Stankov et al., 2014 for a review), there was a moderate/strong positive correlation between the Gf and Confidence factors (*r* = 0.62, *p* < 0.001), a small/moderate positive correlation between the monitoring confidence and control threshold factors (*r* = 0.31, *p* < 0.01), and a weak, non-significant correlation between the Gf and control threshold factors (*r* = -0.19, *p* = 0.07). The parameters of this model were constrained for the next step. These results can be seen as dashed lines in **Figure 4**, which shows the results of the final model conducted in Step 2.

#### Step 2

After fixing parameters from the model in Step 1, the H&B tasks were regressed on the Gf, Confidence, and control threshold factors, and their residual correlations were allowed to covary freely. This model fit the data well: χ^2^_53_ = 76.31, *p* = 0.02, χ^2^/df = 1.44, CFI = 0.97, TLI = 0.96, RMSEA = 0.04 [0.02, 0.06], SRMR = 0.05. However, before discussing the results further, it was notable that not one of the residual correlations between the H&B tasks was significantly greater than zero. Thus, a final model step was taken.

#### Step 3

This final step was done to determine whether the non-significant residual correlations observed between the H&B tasks could be considered equivalent to zero as a whole. As such, the model described in Step 2 was further limited by constraining all of these residual correlations to zero. The model fit was good (χ^2^_63_ = 86.44, *p* = 0.03, χ^2^/df = 1.37, CFI = 0.97, TLI = 0.97, RMSEA = 0.04 [0.01, 0.06], SRMR = 0.06) and, importantly, this constrained model did not differ significantly from the model defined in Step 2 (χ^2^_10_ = 10.13, *p* = 0.43). Thus, the results demonstrated that all of the residual correlations among the H&B tasks were statistically equivalent to zero. This provided sound evidence in favor of Hypothesis 3: that the residual H&B correlations would be weak and, in this case, null. That is, all covariance among the H&B tasks could be explained by cognitive abilities, monitoring, and control. **Figure 4** shows the results for this final model.

All H&B tasks were significantly predicted by one or more of Gf, monitoring confidence, or the control thresholds. Notably large proportions of variance were explained in the Cognitive Reflection Test (*R*^2^ = 0.56) and Applying Decision Rules (*R*^2^ = 0.44). Low proportions of variance were accounted for in the Consistency in Risk Perception (*R*^2^ = 0.07), Resistance to Framing (*R*^2^ = 0.05), and Risky Gambles (*R*^2^ = 0.07).

As hypothesized, individuals with higher Gf (Hypothesis 1a) did better on the performance-based H&B tasks than individuals with lower Gf. Specifically, individuals with higher Gf performed significantly better on all performance-based H&B tasks (all tasks except Risky Gambles). The positive prediction of Gf was clearly stronger for the Cognitive Reflection Test and Applying Decision Rules. These strong and positive predictions were most likely because these tasks are known to require considerable mental processing even after overcoming heuristic responses. The Consistency in Risk Perception and Resistance to Framing tasks, however, do not depend on such a high level of mental processing, hence their lower relation with Gf.

Supporting Hypothesis 1b, lower confidence individuals also performed better than higher confidence individuals on all of these tasks, even after controlling for Gf and control thresholds. This effect was significant for all tasks but the prediction of Consistency in Risk Perception. Control threshold, however, were the weakest overall predictor of H&B performance, supporting, but only weakly, Hypothesis 1c. That is, relative to individuals with a lower threshold, individuals with a higher control threshold were performing significantly better on the Cognitive Reflection Test. In support of Hypothesis 2, control thresholds were also a significant negative predictor of preference for risky gambles. That is, the higher an individual’s threshold, the fewer risky alternatives they selected in the gambling task.

## Discussion

The aim of the research presented here was to investigate whether cognitive abilities, monitoring confidence, and control thresholds could explain individual differences in Heuristics and Biases (H&B; Hypotheses 1a, b, and c, respectively) and gambling tasks (Hypothesis 2). We found that performance and preferences (where relevant) in the H&B tasks used here were predicted by fluid reasoning ability (Gf), monitoring confidence, and to some degree by control thresholds. Using traditional correlational analyses and structural equation modeling, consistent with the theory outlined in the Introduction, individuals with higher Gf, lower monitoring confidence and a higher control threshold performed better on the H&B tasks than individuals with lower Gf, higher confidence and a lower threshold. Furthermore, supporting a broader theoretical application, in the Risky Gambles task, individuals who held a lower control threshold also showed a greater bias for selecting risky rather than certain alternatives. Altogether, this research was the first to investigate and demonstrate that differences in cognitive abilities, monitoring confidence, and control thresholds relate closely to the way individuals respond to H&B tasks.

A further novel aspect of our results was the finding that cognitive abilities, monitoring confidence, and control thresholds explained all of the common variability in the H&B tasks (Hypothesis 3). This result was indicated by null residual correlations between the H&B tasks after they had been regressed on Gf, monitoring confidence, and control threshold factors in a structural equation model. This result was further strengthen by the finding that constraining all of these correlations to zero had no significant effect on the model fit. Thus, after accounting for cognitive abilities, monitoring confidence, and control thresholds, only task-specific factors were driving individual differences on these tasks. These results therefore align with much work describing decision-making as the construction and initiation of actions by cognitive abilities, monitoring, and control (e.g., Koriat and Goldsmith, 1996; Ratcliff and Starns, 2013; Ackerman, 2014; White et al., 2015; Jackson et al., 2016a,b). Still, these results should be considered preliminary, and we hope that future studies will build upon these findings by expanding the selection of H&B tasks to confirm them.

### Explaining Error Detection

Nonetheless, the results align with a theoretical stance that extends existing explanations of individuals’ H&B task performance in an important way. Existing explanations propose the involvement of error detection ability (e.g., Stanovich and West, 2008). Here, we proposed that error detection involves monitoring and control processes. Including monitoring confidence and control thresholds into the model suggests that, accounting for intellectual capacities and skills, individuals adopt a particular decision-making strategy that indirectly results in them being more or less likely to detect errors. The importance of this becomes evident considering tasks outside of the H&B literature. By existing theories, individuals should make better or worse decisions in general (e.g., Fischhoff, 2010), as they are capable of detecting whether they are going to make an error. Thus, an individual who performs well on H&B tasks will perform well in other contexts. Extending this to include monitoring confidence and control thresholds suggests that individuals will make better or worse decisions depending on the context. For example, H&B tasks deliberately elicit the use of heuristics that lead to an incorrect response. Individuals do best on these tasks if they hold low confidence and a high threshold because it gives them time to engage Type 2 processes. However, it is possible to develop highly effective heuristics in other contexts like firefighting (Kahneman and Klein, 2009). In such contexts, the performance of individuals with low confidence and a high threshold will suffer because they will be wasting unnecessary time. The present results, therefore, contribute to the mounting evidence that individuals do not detect errors due to some ability. Rather, individuals who detect more errors have likely adopted a slower, risk-averse strategy, making them best suited for contexts like those faced in H&B tasks.

### Control Unknowns

The one major unknown for this perspective surrounds individual differences in control processes. Compared to cognitive abilities and monitoring confidence, little individual differences research has been conducted on control thresholds. It is possible, for example, that the capacity to set one’s threshold at an optimal point depending on the environment differs between individuals. There is some evidence that this is possible. For example, individuals’ are known to adjust their thresholds differently across contexts (Aminoff et al., 2012; Jackson et al., 2016b). Furthermore, the ability to discriminate accurate from inaccurate judgments via binary decisions differs reliably between individuals (Jackson and Kleitman, 2014; Jackson et al., 2016a,b). Given that monitoring discrimination does not differ between individuals (Stankov and Crawford, 1996; Jackson and Kleitman, 2014), it is possible that differences in thresholds are the cause of this. Overall, control processes are clearly more flexible than monitoring processes. Indeed, it is likely that the relatively weak contribution of control thresholds observed in this study was a result of these domain-specific attributes. It is possible, therefore, that the ability to optimize one’s threshold for a decision task differs among individuals. This notion differs from the traditional concept of error “detection” (which relates more to monitoring) but provides an alternative means by which certain individuals could be better decision makers than others.

### Personality

Replicating previous results, personality variables shared no meaningful relationships with any of the variables under investigations, and were, therefore, omitted from any discussions in this manuscript. We recommend that if timing is an issue and on-task measures of decision-making and confidence are employed, future studies omit personality measures from their research protocol.

### Implications

Additional to these theoretical considerations, considering decision-making in terms of cognitive abilities, monitoring confidence, and control thresholds carries important applied implications. Consider, for example, that individuals of comparable cognitive abilities might be better or worse decision makers depending on the context. Organizations should, therefore, select individuals for very specific decision-making tasks based on their monitoring confidence and control thresholds. Further implications for decision training exist. Certain training programs have adopted H&B tasks as a benchmark for competent decision-making (e.g., Jacobson et al., 2012). Given the results here, it is possible that such training might be encouraging individuals to adopt a particular decision strategy that will only suit a narrow range of contexts. Using H&B tasks for selection or a benchmark of training efficacy should, therefore, be conducted with caution. Good performance on H&B tasks might indicate poorer decision-making in other contexts.

The topic of training is particularly interesting for these reasons. On one hand, the notion of having to train people for particular decision environments is alarming, as it complicates and limits the efficacy of training programs. On the other hand, the possibility that individuals are capable of optimizing their control thresholds opens new avenues. Cognitive processes are notoriously resistant to intervention (Harrison et al., 2013). Monitoring has proven to be slightly more amenable to change (de Bruin and van Gog, 2012; Kleitman and Costa, 2014). Control thresholds are already known to be more flexible and set by our perceptions of payoffs (e.g., Koriat and Goldsmith, 1996; Aminoff et al., 2012; Ackerman, 2014; Jackson et al., 2016b). It is thus likely to be a prime target for intervention. For this reason, we see the potential for training decision control to adapt optimally to different environments as a novel and exciting prospect.

### Limitations

Our results and these implications are not without their limitations, however. As mentioned, we did not explicitly test the error detection ability versus strategy hypothesis. Our findings do contribute to accumulating evidence in favor of the strategy perspective, but these contrasting hypotheses will need to be examined concurrently to make firm conclusions. Should such work be undertaken, we’d advise that researchers take care to dissociate the constructs of importance. For example, thinking-style measures tapping individuals’ tendency to think rationally is often seen as valuable, but this tendency could simply be a product of high control thresholds. It will be useful in future research to concurrently capture a range of constructs related to error detection and compare how they relate to tasks that are representative and non-representative of the environment.

Another general limitation of our results is the use of a student population. On one hand, this has been a consistent element of our previous research (Jackson et al., 2016a,b). We have thus been able to formulate robust expectations about undergraduate students and feel confident that we are tapping into reliable constructs. Still, students differ from the general population on some important attributes that will require investigation. For example, Type 1 knowledge structures and heuristics tend to be acquired over time via extensive learning (Stanovich et al., 2008; Weller et al., 2012). Older populations might, therefore, generate Type 1 responses more frequently and with greater confidence than younger populations. Similarly, university students might generate Type 1 answers more frequently and confidently than young children. It is also important to bear in mind that university students differ from the general population on attributes like cognitive abilities, and our sample were not incentivized to complete the tasks appropriately. Thus, although replicable within a university population, researchers will need to examine the results found here in other age groups or for individuals with different cognitive abilities.

Focal to the results at hand, the domain specificity of the control threshold might be a cause for its relatively weak contribution compared to cognitive abilities and monitoring confidence. Strong domain specificity in control thresholds has been shown to exist (Jackson et al., 2016b). The tests used to derive control thresholds imposed some loss for decision errors. For example, incorrect treatment of a patient in the MDMT results in patient death. There are no penalties, however, for incorrect answers on the H&B tasks. It is thus possible that individuals’ control thresholds in the H&B tasks differed from their thresholds in the other tasks, making the total contribution of control thresholds relatively weak. We suspect that capturing control thresholds in tasks with similar outcomes to the H&B tasks will yield stronger results.

Finally, although we endeavored to collect a broad range of H&B tasks, we were of course limited from a logistical point of view. Our selection of tests did not represent a great many H&B tasks. It will likely be important to test our hypotheses using a different range of H&B tasks. To add to this, we would also recommend that researchers consider the role of cognitive abilities, monitoring confidence, and control thresholds in other decision tasks. Our position is that these three constructs are involved quite generally in the decision-making process. Over 170 tasks are listed in the Decision-Making Individual Differences Inventory (Appelt et al., 2011). It will be important to discern which of these are valid, general and useful for studying decision-making and applying the research findings. We suspect that measures of cognitive abilities, monitoring confidence and control thresholds will yield strong results in such investigations.

## Conclusion

Overall, the results here indicated that individuals’ performance on a range of H&B tasks could be predicted, at times substantially, by the strength of their cognitive abilities (Gf) and their levels of monitoring output (Confidence) and decision control threshold. Furthermore, these constructs explained almost all of the shared covariance among the H&B tasks. The results contribute to the growing concept that cognitive abilities, monitoring, and control are crucial and general psychological mechanisms involved in decision behavior. This concept leads to an important prospect: that after their cognitive capacities, individuals might not possess varied decision skills, but rather adopt varied strategies suited to different contexts. This prospect has important theoretical and applied implications given that H&B tasks are often used to measure benchmark decision performance, but might not indicate universally better decision competence. This skill versus strategy question remains an empirical question. However, it will be an important one to answer for the useful progression of the theory and applied uses of decision sciences. This research adds to previous work finding that cognitive abilities, monitoring confidence, and control thresholds are among the strongest predictors of individual differences in decision-making.

## Ethics Statement

Ethics approval was granted by the University of Sydney Human Research Ethics Committee (project approval number 2012/777). Regarding the procedure, Participants read an information statement describing the research project. They then received a consent form detailing their role and rights as a voluntary participant in the project. They were then able to choose to consent to take part or not.

## Author Contributions

SJ conducted this study as part of a Ph.D. thesis, and thus led the project. Remaining authors provided supervision and ongoing advice regarding all aspects of the manuscript.

## Conflict of Interest Statement

The authors declare that the research was conducted in the absence of any commercial or financial relationships that could be construed as a potential conflict of interest.

## Footnotes

1 Some readers may link control threshold to the criterion of Signal Detection Theory. However, there are aspects of Signal Detection Theory that do not integrate with the theory and prior research on which this research is based. This includes, but is not limited to, the distributional assumptions about signal variance imposed by Signal Detection Theory. For more details, please see our previous work (Jackson et al., 2016a,b) as well as other related papers (Koriat and Goldsmith, 1996; Maniscalco and Lau, 2012; Ratcliff and Starns, 2013; Fetsch et al., 2014). For these reasons, we will refrain from discussion of Signal Detection Theory.

2 In addition to the described tests, participants completed a range of executive functioning measures (e.g., Stroop). However, the executive functioning measures were not usable due to a technical fault in the recording of response times in a web browser, thus we omit them from further reporting.
